# Case report and management approach in idiopathic pulmonary arteries aneurysm

**DOI:** 10.1186/s13019-018-0791-9

**Published:** 2018-10-26

**Authors:** Saleem Haj-Yahia, Mohammad Sbaih, Khalil Bali, Ahmad Darwaze, Wafiq Othman, Mahmoud Zaghari, Gianni Angelini, Massimo Caputo, Abdel-karim Barqawi

**Affiliations:** 1 An-Najah National University Teaching Hospital, Nablus, Palestine; 2 The National Heart and Lung Institute, Nablus, Palestine; 3 Palestine Medical Complex, Ramallah, Palestine; 4 Head of Cardiac Anesthesia Department, Head of Emergency Department, An-Najah National University Teaching Hospital, Nablus, Palestine; 5 Cardio Thoracic Surgery, University of Bristol-United Kingdom, Bristol, United Kingdom; 6 Pediatric Cardiac Surgery, University of Bristol-United Kingdom, Bristol, United Kingdom

**Keywords:** Pulmonary artery, Idiopathic, Aneurysm, Aorta, Lung, Pulmonary valve

## Abstract

**Background:**

Idiopathic pulmonary artery aneurysm is a rare anomaly with only a handful reports in the literature. It is often asymptomatic, while the first presentation could be severe hemoptysis or death.

Surgical intervention needs to be planned carefully with a multidisciplinary team approach to secure optimal outcome**.** We hope to spread our experience with such cases and to encourage other surgeons worldwide to deal surgically with these cases when its indicated.

**Case presentation:**

A 47 years old man presented to our institution after three episodes of hemoptysis, echo demonstrated good left ventricle (LV) systolic function, normal right ventricle (RV) size and function, Chest computed tomography (CT) revealed aneurysmal dilatation with pending rupture of the pulmonary artery trunk (4.5 cm), the left pulmonary artery (6 cm) and the right pulmonary artery (2.3 cm). The patient successfully underwent replacement of Pulmonary artery trunk, left pulmonary artery and right pulmonary artery by Wovex Prosthetic graft (28 mm). The patient discharged home on the eight postoperative day in good clinical condition .

**Conclusion:**

With this case report we wish to emphasize the need for a careful multidisciplinary approach given the complex and rare nature of the reported pathology.

**Electronic supplementary material:**

The online version of this article (10.1186/s13019-018-0791-9) contains supplementary material, which is available to authorized users.

## Background

Aneurysms of the pulmonary artery are a very rare clinical entity with only a handful reported worldwide. Autopsy reported 8 cases of idiopathic pulmonary artery aneurysms (PAAs) out of 109,571 cases [1] with only 4 cases diagnosed and surgically treated. The etiology and pathogenesis have never been clarified completely.Varied clinical presentations of pulmonary artery aneurysm have been reported, though often it is asymptomatic while the first presentation could be hemoptysis or death. Massive and even fatal hemoptysis is the most frequent symptom reported, it occurs in 20–60% of cases [2]. Definitive and clear therapeutic and surgical guidelines are not available. Here we report a large pending rupture idiopathic pulmonary aneurysm with involvement of the main pulmonary artery and all major branches.

## Case presentation

**Fig. 1:**
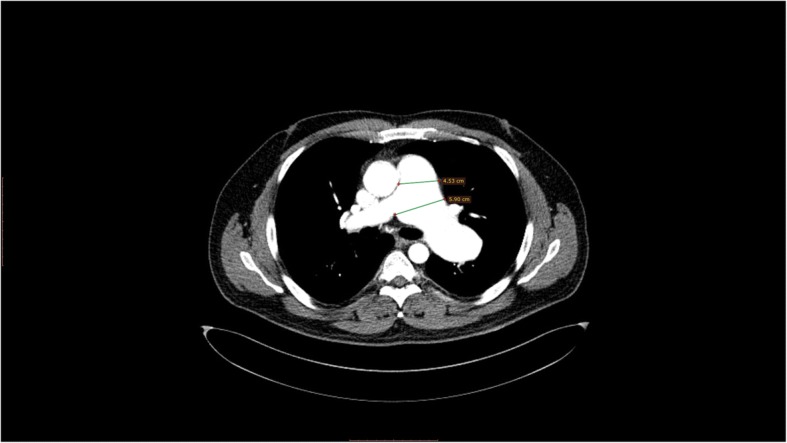
Chest computed tomography angiogram scan (CT) showing: Dilated pulmonary trunk (4.5 cm), Left main pulmonary artery (6 cm), Right main pulmonary artery (2.3 cm)

**Fig. 2:**
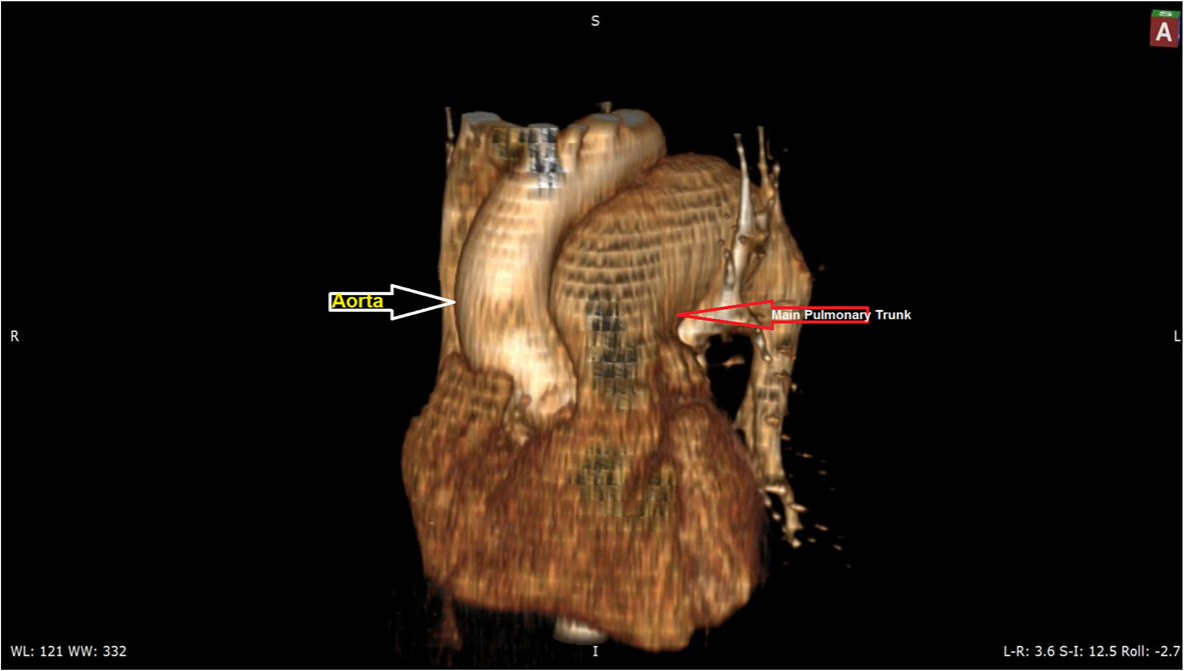
3D - Chest computed tomography angiogram scan (CT) showing: Hugely dilated pulmonary trunk compressing the adjacent Aorta

A 47 years old man presented to our institution after three episodes of hemoptysis. The past medical and family history and Physical examination were unremarkable. Chest X-ray showed wide mediastinum and prominent pulmonary conus. A transthoracic echocardiography showed normal LV systolic function with ejection fraction of 60%, no valve lesion, normal RV size and function and no pulmonary hypertension (PHT) (Additional file 1: Video). Chest computed tomography scan (CT) revealed aneurysmal dilatation with pending rupture of the pulmonary artery trunk (4.5 cm), the left pulmonary artery (6 cm) and the right pulmonary artery (2.3 cm) (Figs. 1 and 2). Cardiac catheterization showed normal coronaries with no signs of compression by the adjacent pulmonary artery aneurysms (PAAs). Acquired causes of pulmonary artery aneurysm, such as syphilis, tuberculosis, Behcet’s disease, Marfan syndrome and vasculitis of pulmonary artery, were all ruled out.

## Surgical technique

**Fig. 3:**
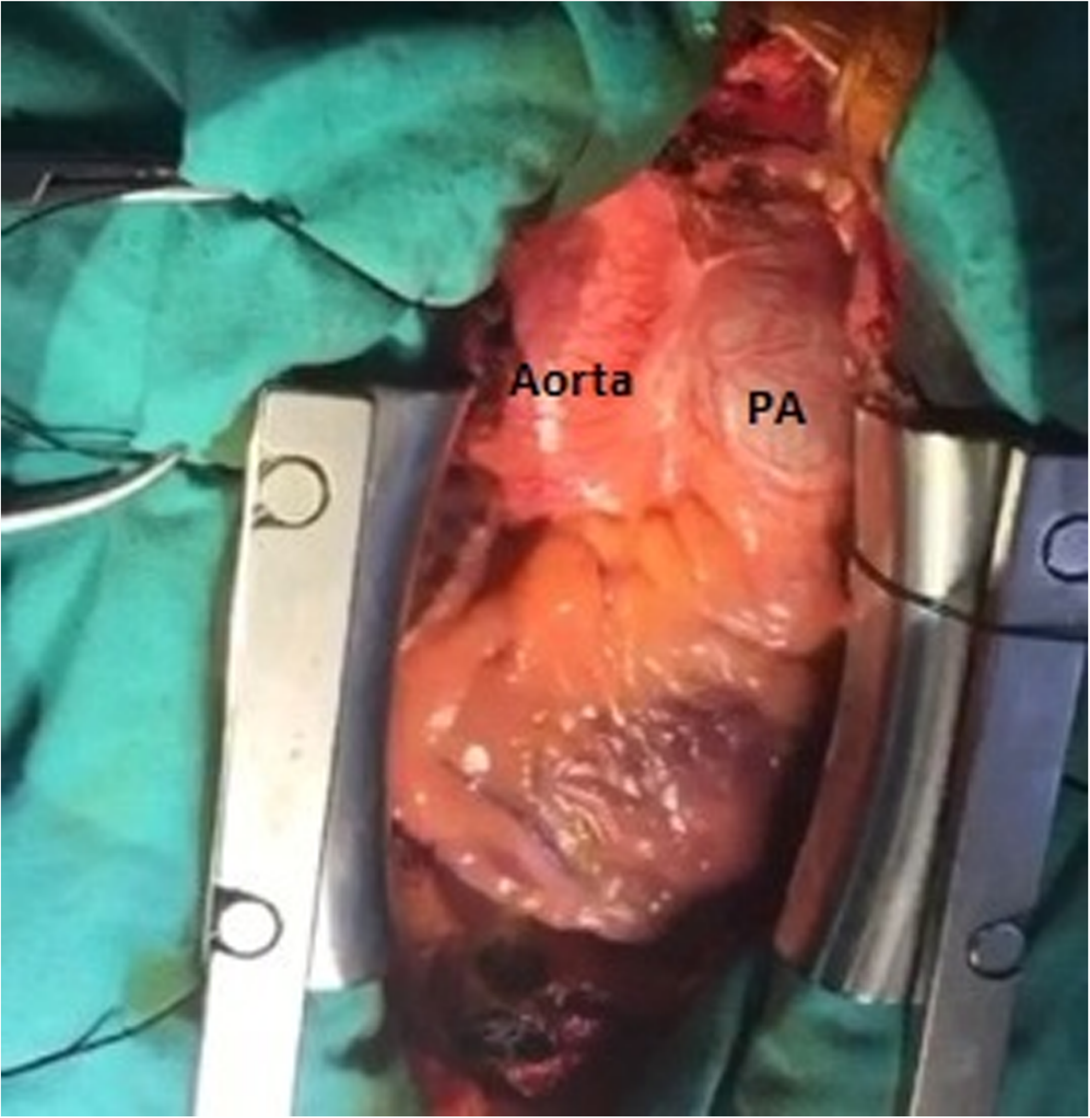
Intraoperative photograph

The surgery was carried out through a median sternotomy. Once we opened the pericardium, a huge pulmonary artery trunk, compressing the adjacent structures, was noticed (Fig. 3). Cardiopulmonary bypass was instituted through bicaval cannulations and hypothermic (temperature 18 c), low flow bypass was established. The main, intraparanchymal left pulmonary artery deep in the left hilum and origin of right pulmonary arteries were fully dissected. They were very frail and even translucent to blood. The dissected edges were strengthened by circumferential pericardial patch. Left pulmonary artery was replaced with 28-mm Wovex tube graft, and the right end of the graft was anastomosed end to end to the origin of right pulmonary artery. The remaining main pulmonary artery was anastomosed end to side to the graft using 4/0 proline.

**Fig. 4:**
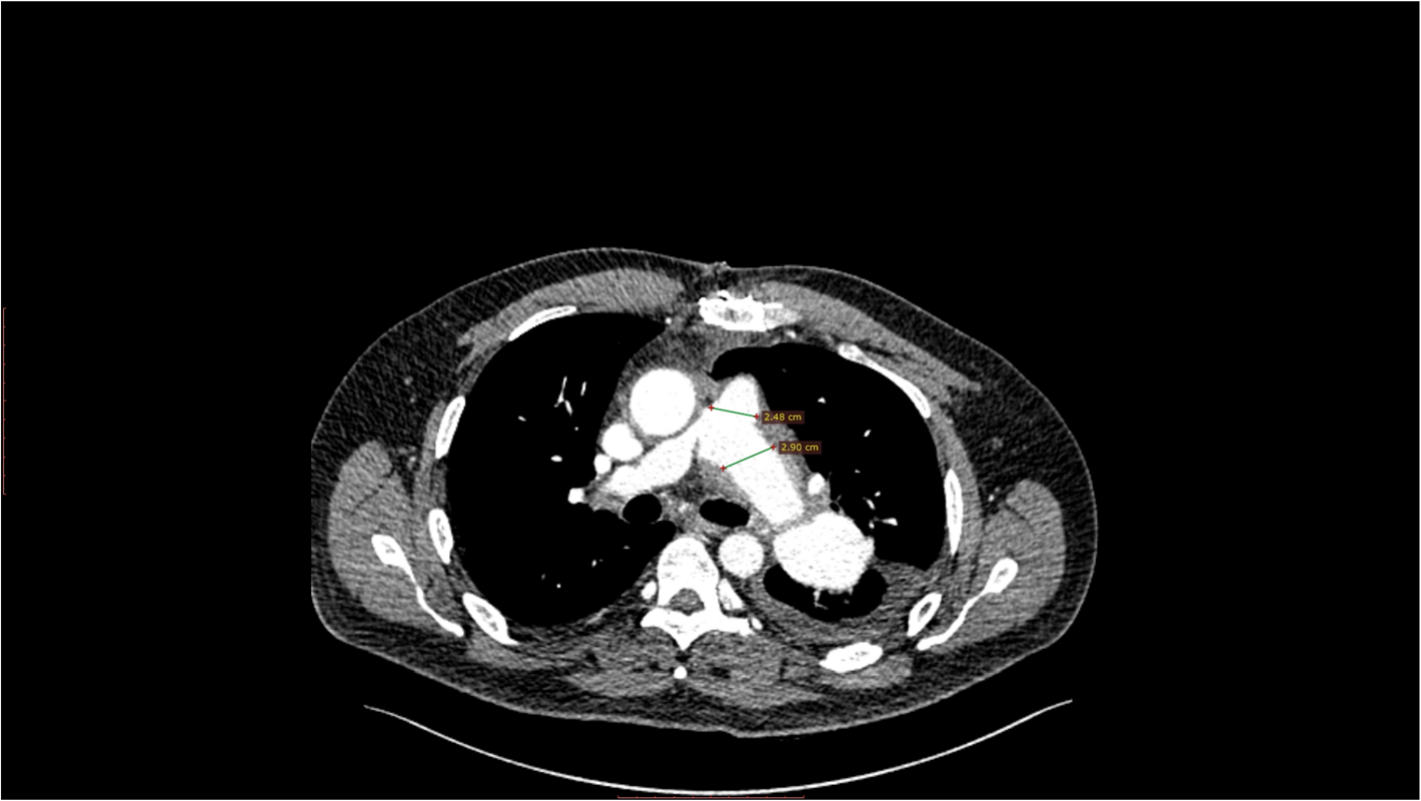
Chest computed tomography angiogram scan (CT) showing: Pulmonary artery trunk, left pulmonary artery and right pulmonary artery replaced by Wovex tube graft without dilatation in the great vessels or stenosis

**Fig. 5:**
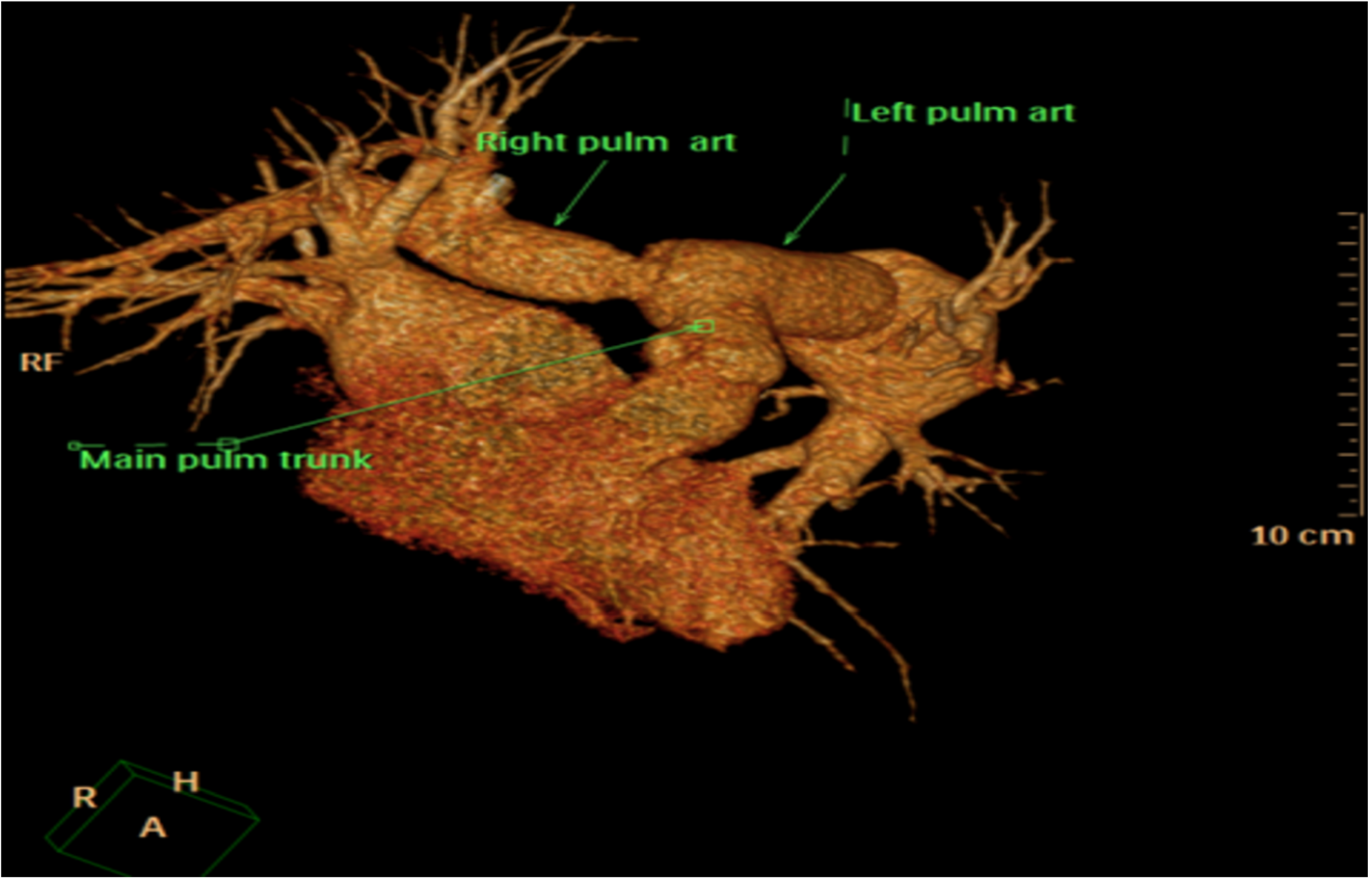
3D - Chest computed tomography angiogram scan (CT) showing: Pulmonary artery trunk, left pulmonary artery and right pulmonary artery replaced by Wovex tube graft without dilatation in the great vessels or stenosis

The early post operative course was uneventfull. The duration of ventilation and ICU stay was 1 and 3 days respectively. The postoperative echocardiogram showed normal RV size and function with no PHT (Additional file 1: Video). On the eight postoperative day and a predischarge chest computed tomography angiogram revealed a normal structures (Figs. 4 and 5). Additionally, right sided heart catheterization was conducted and demonstrated normal pulmonary artery pressure and gradient. During regular followup and after one year from surgery the transthoracic echo was performed and demonstrated normal RV size and function, no valve lesion, normal LV systolic function with ejection fraction 60% and no PHT (Additional file 1: Video).

## Discussion

Our patient fit into the diagnosis of idiopathic pulmonary artery aneurysm, and he fulfilled the standard criteria [3]: there was involvement of the pulmonary artery, absence of abnormal cardiac or extra-cardiac shunts and absence of cardiac or pulmonary disease. These criteria represent the cornerstone for the diagnosis of such cases.

There have been a limited number of cases of idiopathic pulmonary artery aneurysms reported, while the number of surgically treated cases is handful. The surgery is suggested when the patient become symptomatic, pulmonary artery diameter exceeded 5 cm [4, 5].

The most common complications include pulmonary artery dissection and rupture, airway compression, and thrombus in pulmonary artery. On the other hand, conservative treatment is advocated when there is no left-to-right cardiac shunt or significant pulmonary hypertension [6].

The challenges associated with surgery referred to lack of surgical reports. The surgical procedure is highly challenging due to difficulty in accessing the aneurysm, which usually distorts most of the mediastinum. In addition, this is a highly frail tissue which requires special careful handling techniques.

## Conclusion

With this case report we wish to emphasize the need for a careful multidisciplinary approach given the complex and rare nature of the reported pathology.

## Additional file

Additional file 1: Short video demonstrating the intra-operative findings, the surgical technique and the final outcome. (AVI 77905 kb)

## Abbreviations

CT: Chest computed tomography
LV: Left ventricle
PAAs: Pulmonary artery aneurysms
PHT: Pulmonary hypertension
RV: Right ventricle
