# Correction to: Case report and management approach in idiopathic pulmonary arteries aneurysm

**DOI:** 10.1186/s13019-018-0822-6

**Published:** 2019-01-08

**Authors:** Saleem Haj-Yahia, Mohammad Sbaih, Khalil Bali, Ahmad Darwazah, Wafiq Othman, Mahmoud Zaghari, Gianni Angelini, Massimo Caputo, Abdel-karim Barqawi

**Affiliations:** 1 An-Najah National University Teaching Hospital, Nablus, Palestine; 2 The National Heart and Lung Institute, Nablus, Palestine; 3 Palestine Medical Complex, Ramallah, Palestine; 4 Head of Cardiac Anesthesia Department, Head of Emergency Department, An-Najah National University Teaching Hospital, Nablus, Palestine; 5 Cardio Thoracic Surgery, University of Bristol-United Kingdom, Bristol, United Kingdom; 6 Pediatric Cardiac Surgery, University of Bristol-United Kingdom, Bristol, United Kingdom

**Correction to: J Cardiothorac Surg**

**https://doi.org/10.1186/s13019-018-0791-9**

The original article [1] contained an error whereby the author, Ahmad Darwazah’s name was spelt incorrectly. This error has now been corrected.
